# Data describing the effects of depletion of *Myoparr*, *myogenin*, *Ddx17*, and *hnRNPK* in differentiating C2C12 cells

**DOI:** 10.1016/j.dib.2019.104172

**Published:** 2019-06-24

**Authors:** Keisuke Hitachi, Kunihiro Tsuchida

**Affiliations:** Division for Therapies Against Intractable Diseases, Institute for Comprehensive Medical Science (ICMS), Fujita Health University, 1-98 Dengakugakubo, Kutsukake-cho, Toyoake, Aichi 470-1192, Japan

**Keywords:** Skeletal muscle differentiation, RNA-binding protein, transcriptional regulation, RNA-seq, RNA splicing

## Abstract

*Myoparr* is a promoter-associated long non-coding RNA (lncRNA) that is expressed from the promoter region of *myogenin* gene. *Myoparr* is essential for the proper differentiation of skeletal muscle cells; it accomplishes this by activating the expression of *myogenin* and myogenic microRNAs (miRNAs). In this study, we provide the RNA-seq data describing the changes in gene expression induced by knockdown of *Myoparr*, *myogenin*, and two *Myoparr*-binding proteins (Ddx17 and hnRNPK) during skeletal muscle differentiation in C2C12 cells. Raw data files were deposited in Sequence Read Archive in DNA Data Bank of Japan (DDBJ) under the accession number DRA005527. These data are related to the research article “*Myogenin* promoter-associated lncRNA *Myoparr* is essential for myogenic differentiation” Hitachi et al., 2019.

**Table:** Specifications table

Subject area	*Biology*
More specific subject area	*Skeletal muscle differentiation*
Type of data	*Table*
How data was acquired	*High-throughput RNA sequencing using Illumina HiSeq 1500*
Data format	*Raw and analyzed*
Experimental factors	*C2C12 myoblast cells were treated with siRNA and induced to differentiate into myotubes. Twenty-four hours after the induction of differentiation, RNA was extracted, and RNA-seq libraries were generated.*
Experimental features	*C2C12 cells treated with control, non-target siRNA vs. C2C12 cells treated with siRNAs against Myoparr, myogenin, Ddx17, or hnRNPK.*
Data source location	*Toyoake, Aichi, Japan*
Data accessibility	*All Data are available with this article. The RNA-seq raw data were deposited in DDBJ Sequence Read Archive (**http://trace.ddbj.nig.ac.jp/dra/index_e.html**) under the accession number DRA005527 (**http://ddbj.nig.ac.jp/DRASearch/submission?acc=DRA005527**).*
Related research article	*K. Hitachi, M. Nakatani, A. Takasaki, Y. Ouchi, A. Uezumi, H. Ageta,* et al.*, Myogenin promoter-associated lncRNA Myoparr is essential for myogenic differentiation, EMBO Rep. (2019) e47468.**https://doi.org/10.15252/embr.201847468**.**[1]*

**Table:** 

**Value of the data** • The data will be useful for comparing the knockdown effects of promoter-associated lncRNA, *Myoparr*, and its host gene, *myogenin*, in C2C12 cells 24 h after differentiation induction. • Analysis of changes in gene expression induced by the knockdown of lncRNA and its binding-partners (Ddx17 and hnRNPK) will help investigate the importance of binding-proteins in lncRNA function in C2C12 cells 24 h after differentiation induction. • Ddx17 and hnRNPK are also involved in the regulation of RNA splicing in several type of cells [2], [3], [4]. Thus, the data can be further examined to investigate the function of these proteins in RNA splicing processes in C2C12 cells 24 h after differentiation induction.

## 1 Data

The data were deposited in DDBJ Sequence Read Archive (http://trace.ddbj.nig.ac.jp/dra/index_e.html) under the accession number DRA005527. Table 1 shows sample information including individual DDBJ accession IDs. Table 2 represents the number of reads and alignment efficiency. Table S1, Table S2, Table S3, and Table S4 are a list of tables representing all gene expression changes in C2C12 cells treated with siRNAs against *Myoparr*, *myogenin*, *Ddx17*, or *hnRNPK*, respectively.

**Table 1:** The list of sample names and accession numbers of RNA-seq analysis.

Sample information		
Sample name	Sample description	DDBJ accession IDs
Cont-1	Control siRNA transfected, replicate 1	DRR086984
Cont-2	Control siRNA transfected, replicate 2	DRR086985
*Myoparr* KD-1	*Myoparr* siRNA transfected, replicate 1	DRR086986
*Myoparr* KD-2	*Myoparr* siRNA transfected, replicate 2	DRR086987
*myogenin* KD-1	*myogenin* siRNA transfected, replicate 1	DRR086988
*myogenin* KD-2	*myogenin* siRNA transfected, replicate 2	DRR086989
*Ddx17* KD-1	*Ddx17* siRNA transfected, replicate 1	DRR086990
*Ddx17* KD-2	*Ddx17* siRNA transfected, replicate 2	DRR086991
*hnRNPK* KD-1	*hnRNPK* siRNA transfected, replicate 1	DRR086992
*hnRNPK* KD-2	*hnRNPK* siRNA transfected, replicate 2	DRR086993

**Table 2:** The list of numbers of raw and mapped reads of RNA-seq analysis.

A number of reads and alignment efficiency				
Sample name	Raw reads	Reads after trimming	Aligned reads	Overall alignment rate
Cont-1	20,370,169	20,320,153	19,374,206	95.34%
Cont-2	20,386,184	20,320,782	19,235,134	94.66%
*Myoparr* KD-1	18,101,318	18,052,549	17,241,196	95.51%
*Myoparr* KD-2	18,398,677	18,346,104	17,496,761	95.37%
*myogenin* KD-1	18,495,163	18,449,828	17,671,284	95.78%
*myogenin* KD-2	21,871,002	21,808,312	20,757,810	95.18%
*Ddx17* KD-1	17,310,163	17,267,076	16,536,938	95.77%
*Ddx17* KD-2	19,000,103	18,947,096	18,027,321	95.15%
*hnRNPK* KD-1	19,083,614	19,033,967	18,165,588	95.44%
*hnRNPK* KD-2	20,126,755	20,072,850	19,096,155	95.13%

## 2 Experimental design, materials and methods

### 2.1 Cell culture, RNA extraction, and RNA-seq library construction

The mouse myoblast cell line, C2C12, was grown in Dulbecco's modified Eagle medium (DMEM) supplemented with 10% fetal bovine serum at 37 °C and 5% CO_2_ for 24 h before transfection as previously descried [5]. Cells were transfected with 50 nM Stealth RNAi (Thermo Fisher Scientific, MA, USA) using Lipofectamine 3000 (Thermo Fisher Scientific) according to the manufacturer's protocol; stealth RNAi was used as a negative control (Thermo Fisher Scientific, Med GC duplex) while *myogenin* (Thermo Fisher Scientific, MSS275912) and *hnRNPK* (Thermo Fisher Scientific, MSS205172) were the tests. The target sequence of Stealth RNAi for *Myoparr* is as follows; 5′- GATGGACCCTGTCTGATGCTCTTAA -3'. The target sequence of Stealth RNAi for *Ddx17* was previously described [6] and is as follows; 5′- CACCAACAAGGGCACTGCCTATACT -3'. Twenty-four hours after siRNA transfection, C2C12 cells were induced for myogenic differentiation by replacing the medium with DMEM supplemented with 2% horse serum. Twenty-four hours after induction of differentiation, total RNA was isolated from the cells using ISOGEN II regent (Wako, Japan) according to the manufacturer's protocol.

The intact Poly(A)+ RNA was isolated from 1 μg of total RNA using the NEBNext Poly(A) mRNA Magnetic Isolation Module (New England Biolabs, MA, USA) and used for RNA-seq library construction using the NEBNext Ultra RNA Library Prep Kit for Illumina (New England Biolabs) according to the manufacturer's protocol.

### 2.2 Sequencing and data analysis

The libraries were sequenced using Illumina HiSeq 1500 at Fujita Health University. We sequenced two biological replicates for each sample (100 bp single-end reads). The bcl2fastq 1.8.4 software was used for base calling. The raw data for each sample were deposited in DDBJ Sequence Read Archive (Table 1). Quality control and quality trimming of raw sequences were performed using the FastQC ver. 0.11.3 software with the following command "-Q 33 -t 20 -l 30" (https://www.bioinformatics.babraham.ac.uk/projects/fastqc/). Trimmed reads were mapped to the mouse reference genome (mm10) using the Hisat2 ver. 2.0.5 software with the default parameters [7]. The number of mapped reads was about 95% of the original reads (Table 2). Aligned reads were converted to Bam files and sorted using the SAMtools ver. 1.3.1 software [8]. The expression levels of genes were estimated using HTSeq ver. 0.6.0 software [9] against the Mus_musculus_UCSC_mm10.gtf file with the following optional command "--stranded = no --format = bam”. The statistical analysis of differentially expressed genes was calculated using DESeq2 ver.1.12.4 software with a Wald test [10].
